# A 3.8-V earth-abundant sodium battery electrode

**DOI:** 10.1038/ncomms5358

**Published:** 2014-07-17

**Authors:** Prabeer Barpanda, Gosuke Oyama, Shin-ichi Nishimura, Sai-Cheong Chung, Atsuo Yamada

**Affiliations:** 1 Department of Chemical System Engineering, The University of Tokyo, 7-3-1 Hongo, Bunkyo-ku, Tokyo 113-8656, Japan; 2 Unit of Element Strategy Initiative for Catalysts and Batteries, ESICB, Kyoto University, Kyoto 615-8510, Japan; 3 Materials Research Center, Indian Institute of Science, Bangalore 560012, India; 4 These authors contributed equally to this work

## Abstract

Rechargeable lithium batteries have ushered the wireless revolution over last two decades and are now matured to enable green automobiles. However, the growing concern on scarcity and large-scale applications of lithium resources have steered effort to realize sustainable sodium-ion batteries, Na and Fe being abundant and low-cost charge carrier and redox centre, respectively. However, their performance is limited owing to low operating voltage and sluggish kinetics. Here we report a hitherto-unknown material with entirely new composition and structure with the first alluaudite-type sulphate framework, Na_2_Fe_2_(SO_4_)_3_, registering the highest-ever Fe^3+^/Fe^2+^ redox potential at 3.8 V (versus Na, and hence 4.1 V versus Li) along with fast rate kinetics. Rare-metal-free Na-ion rechargeable battery system compatible with the present Li-ion battery is now in realistic scope without sacrificing high energy density and high power, and paves way for discovery of new earth-abundant sustainable cathodes for large-scale batteries.

## Abstract (web summary)

Cathode materials for sodium-ion batteries often suffer from low operating voltage, sluggish kinetics and high cost. Here, the authors report an iron-based alluaudite-type sulphate cathode, which could achieve a high redox potential of 3.8 V versus sodium, high energy density and fast rate kinetics.

## Graphical abstract

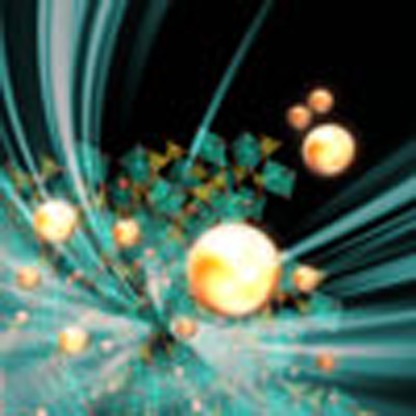

Lithium (Li)-ion battery was intensely explored in 1980s leading to its commercialization in 1990s. Ever since, the synergistic effort in basic science and industrial optimization has led to the doubling of energy density. Currently, Li-ion batteries are ubiquitous in suites of small-scale consumer electronics, power tools as well as large-scale power sources driving the (plug-in) hybrid electric transportation and power-grid systems. The ever-growing global population and the meteoric rise in demand of easy access to modern technologies (gadgets/automobiles) have created multi-billion dollar battery industry. The current generation Li-ion batteries use oxides (such as LiCoO_2_ and LiMn_2_O_4_) and olivine LiFePO_4_ as cathodes^1^,^2^,^3^. Suddenly, this manifold consumption of Li has led to its scarcity and price rise, with many raising a concern whether Li is the new gold that may trigger geo-political tension in future^4^. The vast range of battery applications can be divided into two broad categories: volume/weight-restricted applications such as electronics/automobiles and volume/weight-less-dependent uses such as remote area large power-grid systems for efficient use of electricity transmitted from thermal power plants and solar/wind mills. While the Li batteries are indispensable for former category, the later category has been economically catered in part by Na–S batteries operating at high temperature over 300 °C. Resource optimization and tailor-made battery design for different applications, including dense smart grid with self-management housing system, is a global call, where Na-ion batteries operating at ambient temperatures can have vital role. Contrary to Li, sodium (Na) has abundant natural resources with even geographic distribution. Being the fifth most abundant element in earth’s crust, Na charge carrier is also the second lightest alkali element in periodic table. In this context, mammoth effort has been geared to build efficient Na-ion batteries with optimization of energy density, rate kinetics, low cost as well as safe and sustainable production and operation. In this pursuit, numerous Fe-based cathode compounds capable of efficient Na (de)insertion have been reported^5^,^6^,^7^,^8^,^9^,^10^,^11^.

Looking back to the history, soon after the conceptualization of intercalation reaction into TiS_2_ host in 1976 (ref. 12), research into Li-based and Na-based insertion compounds kick-started in early 1980s (Li_x_CoO_2_ in 1980 (ref. 1) and Na_x_CoO_2_ in 1981 (ref. 5)). However, the commercial prospects of light-weight Li-ion batteries in portable electronics steered massive effort on Li systems, resulting in a two-decade-long hibernation period for Na counterparts. Over the past few years, the renewed interest on Na-ion chemistry has seen extensive research on various P2- and O3-type oxide-layered compounds and their solid solutions, mostly based on expensive transition metals such as Co and Ni. The large-scale Na-batteries will be commercially viable with earth-abundant transition metal such as Fe. Till date, O3-type NaFeO_2_ (ref. 13) and P2-type Na_x_[Fe_1/2_Mn_1/2_]O_2_ (ref. 7) are reported, both suffering from low operating potential even by using Fe^4+^/Fe^3+^ redox couple, and stable reversible capacity is limited. Using the inductive effect in polyanion framework systems, Fe^3+^/Fe^2+^ redox potential can be enhanced with full utilization of one-electron reaction^14^. In this pursuit, many ~3 V Fe-based phosphate PO_4_^3−^ insertion compounds have been reported. They are Na_2_FePO_4_F (3.06 V; ref. 8), NaFePO_4_ (2.7 V; ref. 9), Na_2_FeP_2_O_7_ (3 V; ref. 10) and Na_4_Fe_3_(PO_4_)_2_(P_2_O_7_) (3.2 V; ref. 11). Newer Fe-based compounds with higher electrode potential can be realized by replacing phosphate PO_4_^3−^ with sulphate SO_4_^2−^ units taking advantage of their higher electronegativity^14^. This avenue is not yet realized with the only known SO_4_^2−^-based compounds NaFeSO_4_F and NaFeSO_4_F.2H_2_O being electrochemically inactive^15^,^16^. Herein, we report an entirely new class of cathode, Na_2_Fe_2_(SO_4_)_3_, combining the unusually high Fe redox potential ~3.8 V versus Na with excellent rate kinetics as well as good economy. It benchmarks the highest-ever Fe^3+^/Fe^2+^ redox potential by far observed among all known oxides and oxyanionic insertion materials for Na-ion batteries.

## Results

### Materials synthesis and crystal structure

**Figure 1:**
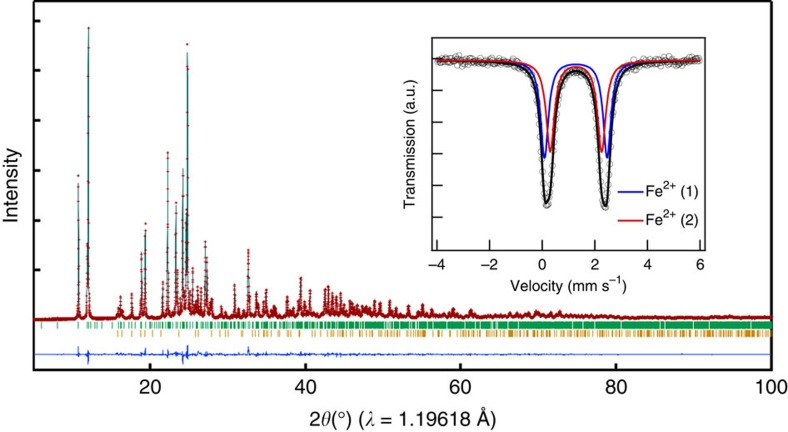
XRD pattern of Na_2_Fe_2_(SO_4_)_3_. Rietveld refinement pattern of powder XRD data for Na_2_Fe_2_(SO_4_)_3_. Experimental data and calculated profile and their difference are shown as red crosses and black and purple solid lines, respectively. The theoretical Bragg positions are shown with green ticks. Trace amount (about 4 wt%) of bata-FeSO_4_ as an impurity was included in the analysis, as indicated by yellow ticks. (Inset) Room temperature Mössbauer spectrum of pristine Na_2_Fe_2_(SO_4_)_3_ shows the existence of two distinctive Fe(II) sites in 1:1 ratio (red and blue lines).

Unlike the oxides and various polyanions (BO_3_^3−^, PO_4_^3−^ and SiO_4_^4−^) compounds, the SO_4_^2−^ containing systems are acutely prone to thermal decomposition above ~400 °C (leading to SO_2_ gas evolution). In addition, inherent dissolution of SO_4_^2−^ in water makes it unstable in aqueous media. It rules out conventional high-temperature solid-state and aqueous solution-based synthetic routes. Thus, we used low-temperature (*T*_r_ ≤350 °C) solid-state methods to obtain Na_2_Fe_2_(SO_4_)_3_ target compound. The unknown crystal structure of this new cathode material was determined by synchrotron powder X-ray diffraction (XRD) (Fig. 1). Rietveld refinement and Mössbauer data (Fig. 1, inset) confirm trace amount of Fe(III) impurity phase(s). Mössbauer spectrum of the pristine material, consisting only Fe(II) species, could be fitted with two doublets having 1:1 intensity ratio, which can be assigned to two distinct crystallographic sites, Fe(1) and Fe(2). All the Bragg reflections were indexed in a monoclinic lattice assuming *P*2_1_/*c* (No. 14) symmetry with lattice parameters *a*=11.46964(8) Å, *b*=12.77002(9) Å, *c*=6.51179(5) Å, *β*=95.2742(4)° and *V*=949.73(1) Å^3^. Although non-stoichiometry was to be considered, the fitting was satisfactory (*R*_wp_=4.87%, *R*_p_=3.94%, *R*_Bragg_=1.58% and Goodness of fit (GoF)=1.74). Trace amount (about 4 wt%) of bata-FeSO_4_ as an impurity was included in the analysis. The crystallographic data are summarized in Supplementary Tables 1 and 2. Indexing and analysis adopting alternative *C2/c* symmetry with one Fe site was also possible with slight increase in *R*_Bragg_ as summarized in Supplementary Tables 3 and 4, indicating that local environments of two Fe sites are quite similar. Although *P2*_*1*_*/c* symmetry seems more suitable in the present analysis, further systematic approach by electron diffraction and/or single-crystal XRD would be necessary for the firm conclusion on *P2*_*1*_*/c* versus *C2/c*.

**Figure 2:**
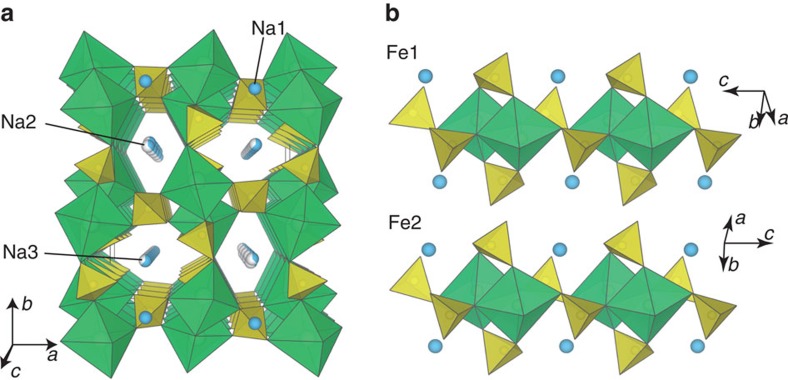
Crystal structure of Na_2_Fe_2_(SO_4_)_3_. (**a**) The structure of Na_2_Fe_2_(SO_4_)_3_ projected along the *c* axis; and (**b**) local environment of two independent Fe sites. Green octahedra, yellow tetrahedra and blue spheres show FeO_6_, SO_4_ and Na, respectively. Fe ions occupy two kinds of crystallographic sites that have distinctive octahedral geometries. Each FeO_6_ octahedra share an edge with the crystallographically equivalent octahedra and form Fe_2_O_10_ dimers. The SO_4_^2−^ anions interconnect these dimers so as to build up a three-dimensional framework structure.

The refined crystal structure of Na_2_Fe_2_(SO_4_)_3_ is shown in Fig. 2a. To the best of our knowledge, the composition and crystal structure of Na_2_Fe_2_(SO_4_)_3_ are completely new and have never been reported in the literature. Deviating sharply from most of the *A*_x_*M*_2_(*X*O_4_)_3_-type compounds adopting the NASICON-related structures, Na_2_Fe_2_(SO_4_)_3_ does not contain the lantern units [*M*_2_(*X*O_4_)_3_], forming a unique structure with alluaudite-type framework. It would be convenient to denote *AA′BM*_2_(*X*O_4_)_3_ as general alluaudite-type compounds, where *A*=partially occupied Na(2), *A′*=partially occupied Na(3), *B*=Na(1), *M*=Fe^2+^ and *X*=S in the present case. To the best of our knowledge, this is the first sulphate compound with alluaudite-type framework. The Fe ions occupy octahedral sites that share edges with a crystallographically equivalent octahedron, forming Fe_2_O_10_ dimer units. These Fe ions were assigned to two distinct crystallographic sites, Fe(1) and Fe(2) (Fig. 2b). Even though the local structures of Fe(1) and Fe(2) are similar to each other, they are crystallographically distinct as revealed by two doublets in the Mössbauer spectrum (Fig. 1, inset). These isolated edge-sharing Fe_2_O_10_ dimers are in turn bridged together by SO_4_ units strictly by corner-sharing mode, hence forming a three-dimensional framework with large tunnels along *c* axis. The constituent Na occupies three distinct crystallographic sites; one fully occupied and two partially occupied. This new structure type should open up an entirely new Na_2−*x*_*M*_2_(SO_4_)_3_ (*M*=Mg, Ti, Mn, Co, Ni, V and VO) family of compounds as potential cathodes/anodes/solid electrolytes for further material exploration. Although NaMnFe_2_(PO_4_)_3_ compounds with alluaudite-type *AA′BM*_2_(*X*O_4_)_3_ framework of *A, A′*=partially occupied Na, *B*=Mn^2+^ and Fe^2+^, *M*=Mn^3+^ and Fe^3+^ and *X*=P was previously synthesized^17^, it showed weak electrochemical reactivity. Great advantage to use (SO_4_)^2−^ instead of (PO_4_)^3−^ is to stabilize the nearly Na–Fe equi-amount Na_2_Fe_2_(SO_4_)_3_ compound including only Fe^2+^ with partially occupied Na^+^ sites suitable for fast Na^+^ diffusion upon electrode reaction.

### Electrochemical properties

**Figure 3:**
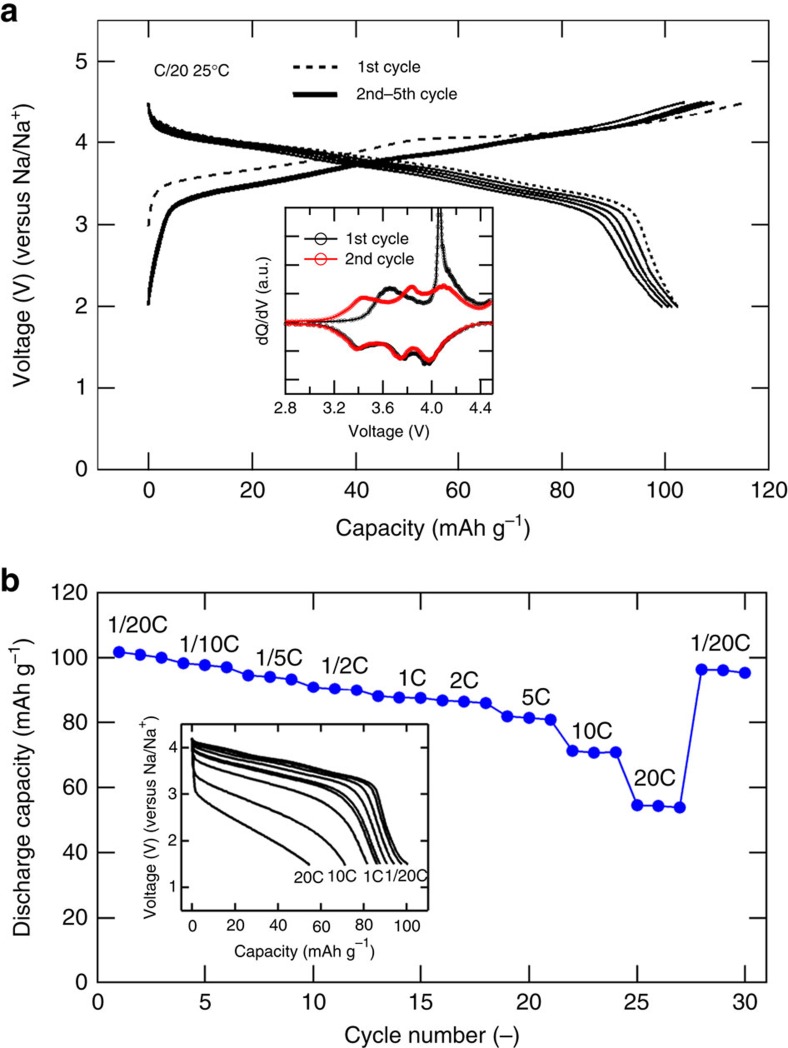
Electrode properties of Na_2−*x*_Fe_2_(SO_4_)_3_ in Na cell. (**a**) Galvanostatic charging and discharging profiles of Na_2−*x*_Fe_2_(SO_4_)_3_ cathode cycled between 2.0 and 4.5 V at a rate of C/20 (2 Na in 20 h) at 25 °C. First (1st) cycle is shown in dashed black line, and 2nd–5th cycle in solid black lines. (Inset) The differential galvanostatic profiles (d*Q*/d*V*) of Na_2−*x*_Fe_2_(SO_4_)_3_ cathode showing two distinctive peaks during the first charge and broader three peaks upon subsequent discharging/charging processes. (**b**) Capacity retention upon cycling up to 30 cycles under various rate of C/20 (2 Na in 20 h) to 20C (2 Na in 3 min). (Inset) The discharge curves of Na_2−*x*_Fe_2_(SO_4_)_3_ as a function of rate (from C/20 to 20C). Before each discharge, the cells were charged at C/10 to 4.2 V.

The electrode properties of as-synthesized Na_2_Fe_2_(SO_4_)_3_ were examined with no further optimization such as particle downsizing or carbon coating. The primary particle size was evaluated to be ~100–200 nm by scanning electron microscope observation (Supplementary Fig. 1), and the electrode loading was *ca*. 3 mg cm^−2^. The corresponding voltage-capacity profiles for first few cycles between 2.0–4.5 V (versus Na/Na^+^) at a rate of C/20 (25 °C) is shown in Fig. 3a. The Na_2_Fe_2_(SO_4_)_3_ cathode offers an average potential of 3.8 V (versus Na/Na^+^), which is the highest-ever Fe^3+^/Fe^2+^ redox potential in any materials environment. The well-known NASICON-type Fe(III)_2_(SO_4_)_3_ (ref. 18) has same composition with desodiated Na_2_Fe_2_(SO_4_)_3_ in the present study, but NASICON phase delivers an average potential of 3.3 V (versus Na/Na^+^) upon Na insertion^19^.

Thereby, Na_2_Fe_2_(SO_4_)_3_ cathode is characterized by three distinctive features: (i) totally new pristine composition with structure with edge-sharing FeO_6_ octahedra different from NASICON- or NASICON-like phases with corner-sharing FeO_6_ octahedra; (ii) initial valence state is Fe(II) with inherent existence of Na in the structure allowing to function as a cathode of Na-‘ion’ battery system; and (iii) much higher electrode potential by ~0.5 V comparing to the NASICON phases, providing very suitable operating potential of 3.8 V (versus Na/Na^+^) with smooth sloppy charge–discharge profiles over a narrow voltage range in 3.3–4.3 V window. Features (i) and (ii) account for the abnormally high potential of Na_2_Fe_2_(SO_4_)_3_ (ref. 20). The voltage *E* can be expressed as *E*=Δ*G*°/*nF*=(*xG*°_Na_+*G*°_Host_−*G*°_Na*x*Host_)/*nF*, where *n*, *F* and *G* are number of electrons, Faraday constant and Gibbs free energy, respectively. For Na_2_Fe_2_(SO_4_)_3_, the difference *G*°_Host_−*G*°_Na*x*Host_ is large since the sodiated state is synthesized as stable state (low *G*°_Na*x*Host_), whereas the desodiated one is electrochemical-generated (possibly metastable) state (high *G*°_Host_). This is reverse for NASICON-type Fe_2_(SO_4_)_3_ and any related Fe(III) cathodes. Another factor, the edge-sharing geometry of the Fe octahedra in Na_2_Fe_2_(SO_4_)_3_, will push up *G*°_Host_ owing to the strong Fe^3+^–Fe^3+^ repulsion, leading to high *E*^19^. This geometric characteristics can be found in other high-voltage materials such as triplite-type LiFeSO_4_F (ref. 21), and Li_2_FeP_2_O_7_ (ref. 22). In fact, Na_2_Fe_2_(SO_4_)_3_ has the shortest Fe–Fe distance among these materials. Thus, the operating potential of 3.8 V by Na_2−*x*_Fe_2_(SO_4_)_3_ (that is, 4.1 V versus Li) records the highest value among all Fe-based battery cathodes; it is even higher than those of Fe^4+^/Fe^3+^ redox couple in simple oxides as Na_1−x_FeO_2_ (Fig. 5). Surprisingly, it exceeds the highest record in Li system in Li_1−x_FeSO_4_F, Li_2_FeP_2_O_7_ and Li_2_Fe(SO_4_)_2_ (~3.9 V versus Li and hence 3.6 V versus Na)^21^,^22^,^23^,^24^. Unlike the fluorosulphate cathodes, this high redox voltage is obtained without using electronegative F^−^ units that make the synthesis cumbersome and enhances hygroscopic/instability in the final cathodes.

The initial reversible capacity of 102 mAh g^−1^, which corresponds to 85% of one-electron theoretical capacity (*ca*. 120 mAh g^−1^) based on Fe^3+^/Fe^2+^ redox couple, was highly reversible over 30 cycles under various current rate (Fig. 3b). Irreversible capacity of <14 mAh g^−1^ (>88% charge–discharge efficiency) in Fig. 3a may come from electrolyte decomposition as the cell was charged up to very high voltage, 4.5 V versus Na (4.8 V versus Li). When the current is further increased, 86% (versus the value at C/20) of the initial capacity can be delivered in 1 h (1C), 85% in 30 min (2C) and 70% in 6 min (10C) as shown in Fig. 3b. This high-rate capability of Na_2−*x*_Fe_2_(SO_4_)_3_ electrode suggests that Na-ion migration in the framework structure is fast, as will be discussed in the later section.

### Reaction mechanisms

In spite of its high power operation and excellent cyclability, the voltage profile of the initial charge was slightly different from those of the subsequent cycles. During the first charge segment, the average redox reaction occurs at 3.9 V (versus Na), which drops to 3.8 V in subsequent cycles. The differential galvanostatic profiles (d*Q*/d*V*) of Na_2−*x*_Fe_2_(SO_4_)_3_ cathode (inset of Fig. 3a) showing two distinctive peaks (3.65 and 4.06 V versus Na/Na^+^) at the first charge and broader three peaks (3.42, 3.80, and 4.04 V in the middle points versus Na/Na^+^) upon subsequent discharging/charging processes. These indicate the occurrence of some irreversible structural transformation during first desodiation process, similar to the cases of Li_2_FeSiO_4_ and Li_2_FeP_2_O_7_ (ref. 22). The sloping voltage curve over the entire range of Na composition suggests a single-phase homogeneous reaction mechanism involving minimal volume change. This hypothesis was verified by comparative XRD patterns and Mossbauer Spectra of the Na_2−*x*_Fe_2_(SO_4_)_3_ (*x*=0–1.6) compositions prepared by chemical oxidation (Supplementary Fig. 2 and Supplementary Table 5), as well as by *in situ* XRD measurement during electrochemical charge–discharge (Supplementary Fig. 3). Continuous shift of diffraction peaks with mere volume change (Δ*V*) of −1.6% was confirmed, and in striking contrast to the Li_x_FePO_4_ system dominated by the two-phase separation^25^. This is beneficial for long-term cycling, uniform reaction over the whole electrode and longevity of the cathode involving less aggressive electromechanical grinding during its operation^26^. Such a small volume change in charge–discharge reaction may give another explanation for the high-rate capability but is quite surprising, considering much larger ionic radius of Na^+^ than that of Li^+^. Indeed, the Δ*V*= −17.6% in Na_*x*_FePO_4_ has been reported to be much larger than Δ*V*= −6.9% in Li_*x*_FePO_4_ (refs 9, 27).

### Na-ion dynamics

**Figure 4:**
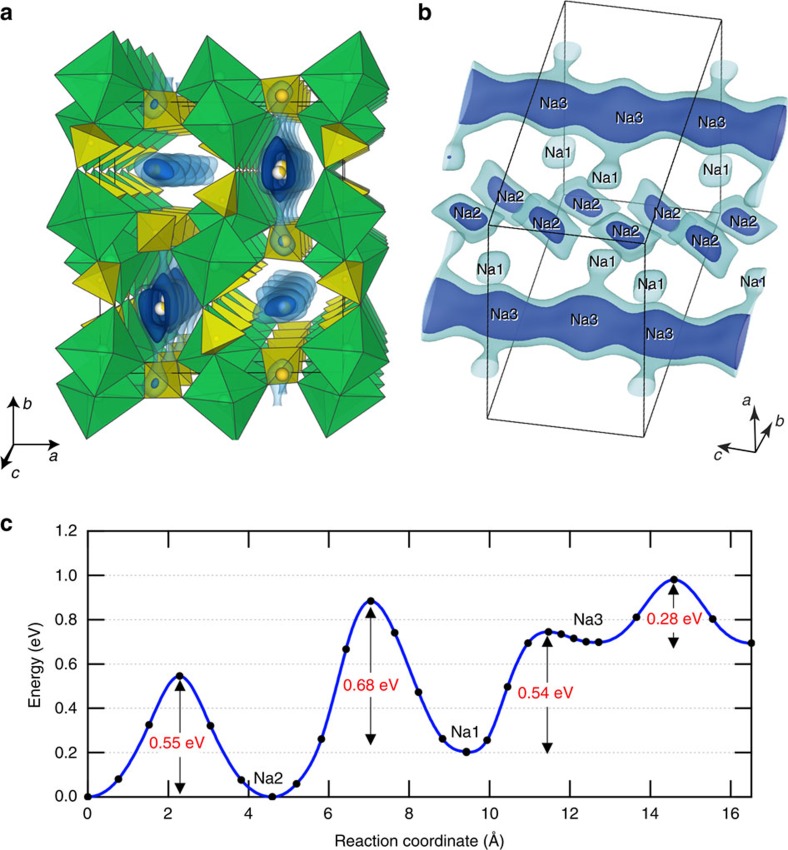
Na-ion diffusion in Na_2_Fe_2_(SO_4_)_3_. (**a**,**b**) Equi-value surface of the Δ*BVS.* The blue and light-blue surfaces are for Δ*BVS*=0.2 and 0.4, respectively. Inner side of the surface corresponds to accessible spaces for the Na ions. Green and yellow polyhedra are that of FeO_6_ and SO_4_, respectively. (**c**) Migration activation energy of Na^+^ ion calculated with DFT. Shown are the values (from left to right) for migrations along the *c* axis for the Na2 sites, between Na2 and Na1 sites, between Na1 and Na3 sites, and along the *c* axis for the Na3 sites. Calculations are done at low concentration of Na.

Na_2_Fe_2_(SO_4_)_3_ turns out to be an ideal host structure for efficient and fast Na^+^ (de)insertion with unusually high Fe redox potential. To gain further insight on this suitable structure, bond valence (BV) method was used to evaluate the validity of the crystal structure as well as to elucidate possible Na diffusion paths by utilizing the soft-BV parameters^28^,^29^. Difference of the BV sum from the ideal value (Δ*BVS*) provides a simple measure of positional suitability of mobile ions in solid frameworks^30^. Figure 4 shows a map of Δ*BVS* as equi-value surface. Inner side of the equi-value surface shows accessible spaces for Na^+^ in the [Fe_2_(SO_4_)_3_]^2–^ framework. All the refined Na positions are consistent with the Δ*BVS* map where the maximum Δ*BVS* at Na positions are <0.2. Although the Na1 and the Na2 looks to have rather localized character in the present analysis, the Na3 site are clearly permeating along the [001] direction.

*Ab initio* calculations were performed to gain more quantitative understandings. The calculations were conducted for the Na poor region (see Methods section for details). At this concentration, we found that the binding energy of Na to the Na2 site is most favourable and to the Na3 least favourable. The activation energies for the Na-ion migration within the Na3 channel indeed is found to be low, which is 0.28 eV. Liquid-like value of 0.14 eV was calculated for defect diffusion along the channel and is among the lowest for Na-ion conductors^31^. The value for the Na2 channel is 0.54 eV similar to that for Na_2_FeP_2_O_7_, which shows very fast charge–discharge kinetics^10^,^32^,^33^. For migrations between channels, the activation energies are 0.88 and 0.58 eV for back-and-forth transport between Na1 and Na2 sites, and they are 0.54 and 0.05 eV for that between Na1 and Na3. We can therefore postulate that this material has one-dimensional Na^+^ conduction channels along the *c* axis for both the Na2 and Na3 sites, while the Na1 ion can be extracted through the Na3 sites. As a result, all the Na ions are accessible for (de)intercalation reaction with no limitation towards theoretical capacity. In particular, the continuous space around Na3 site can act as a fast Na transport channel during the charge–discharge reaction, which can be the origin of excellent kinetics of Na_2_Fe_2_(SO_4_)_3_ cathode material. Similar technical strategies applied for Li_x_FePO_4_, which also shows one-dimensional diffusion^34^, should be effective to enhance electrode performance such as diminishing defect density and minimizing particle size along *c* axis^35^.

### Material stability

Finally, the material stability (chemical/thermal/storage) was examined^36^. Similar to other sulphate-based cathodes (for example, fluorosulphates and bisulphates), Na_2_Fe_2_(SO_4_)_3_ was found to dissolve completely in water. Thus, it is not stable in aqueous condition, a fact that was further verified by observation of its steady degradation upon long-time moisture exposure (in ambient condition) to form a hydrated derivative Na_2_Fe(SO_4_)_2_.4H_2_O. Nevertheless, with minimal exposure of freshly prepared sample to ambient air and careful packaging/storage (in inert atmosphere), this metastable compound remains intact with no deterioration in its electrochemical properties. Further, the thermal analysis of Na_2_Fe_2_(SO_4_)_3_ noticed gradual weight loss upon heating above 450 °C with simultaneous decomposition of SO_4_ units, release of SO_2_ gas and oxidation of Fe^2+^ species leading to the formation of Na_2_SO_4_, Fe_2_O_3_ and Fe_3_O_4_. In spite of this thermal decomposition above 450 °C, it should be noted that Na_2_Fe_2_(SO_4_)_3_ compound offers sufficient thermal stability of real-life battery applications.

## Discussion

**Figure 5:**
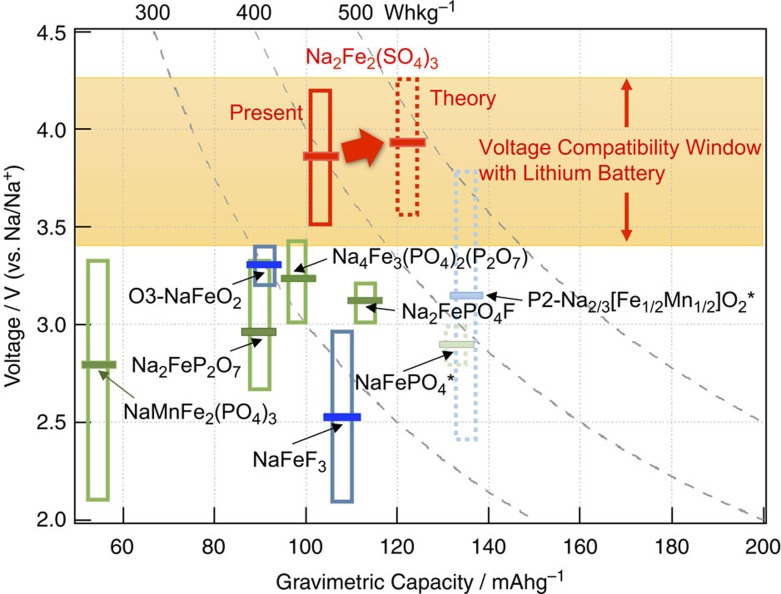
Overall comparison of the Fe-based cathode materials that can function as Na sources in Na-ion battery system. Polyanionic cathode materials are shown as green boxes and simple oxides/fluorides as blue, respectively. Horizontal bars represent average voltage. Yellow band indicates voltage region that can ensure the compatibility with Li-ion batteries. The new compound Na_2_Fe_2_(SO_4_)_3_ is presented by the red box together with its expected dashed-red region based on the theoretical capacity. (*The capacity and voltage of P2-Na_2/3−x_[Fe_1/2_Mn_1/2_]O_2_ is assumed by 0<*x*<2/3 region by inherent amount of Na with large hysteresis including both Fe^4+^/Fe^3+^ and Mn^4+^/Mn^3+^ redox reactions, as separately denoted with dashed pale blue box. Dashed pale green box for NaFePO_4_ indicate it cannot be directly synthesized and sluggish kinetics in electrode reaction.)

Searching for novel low-cost cathode materials for rechargeable Na-ion batteries, we have synthesized a whole new family of cathode materials with general formula Na_2_*M*_2_(SO_4_)_3_. The first such candidate, Fe-based Na_2_Fe_2_(SO_4_)_3_, delivers a reversible capacity exceeding 100 mAh g^−1^ with the working Fe^3+^/Fe^2+^ potential located at 3.8 V (versus Na/Na^+^), the highest known value among all Fe-based insertion compounds. This abnormally high voltage is compatible with the thermodynamic limit of current generation organic electrolytes offering stable/safe operation. In addition, it offers excellent rate kinetics and cycling stability without demanding any additional cathode optimization. It forms an open framework host for efficient (de)intercalation of Na ions with very low activation energy. Operating voltage and reversible capacity of various known iron-based cathode for Na-‘ion’ (cathode functions as whole Na source) battery system are summarized in Fig. 5. The new material Na_2_Fe_2_(SO_4_)_3_ is benchmarking and worth further optimizing as it is the first Fe-based cathode for Na battery to offer high voltage compatible with Li battery system. Moreover, further effort to reach theoretical capacity by full utilization of inherent Na ions (85% in the present paper) can lead to energy density of >540 Wh kg^−1^, which is higher than those of LiMn_2_O_4_ (430 Wh kg^−1^) and LiFePO_4_ (500 Wh kg^−1^).

Complementing this electrode performance, Na_2_Fe_2_(SO_4_)_3_ can be easily prepared and upscaled by low-temperature solid-state methods, although care should be taken on the hygroscopic nature. The sustainability of Na_2_Fe_2_(SO_4_)_3_ further arises from its economic Na–Fe–S–O elemental constitution. In earth’s upper crust, Na and Fe are the most abundant and geographically distributed alkali and 3*d* transition metal, respectively. Talking about sulphur and sulphate compounds, they are very economic and widely used in fertilizers, pesticides and chemical industries. In fact, they are extremely cheap, being a byproduct of fuel combustion, coal power plants and oil/petrochemical industries. Thus, Na_2_Fe_2_(SO_4_)_3_ form an ideal material for economic production and large-scale battery manufacturing. We strongly believe that Na_2_Fe_2_(SO_4_)_3_ cathode will not only open up a new sub-group of polyanionic cathodes with commercial potential, but also inspire future success in discovering superior electrode materials for next-generation secondary batteries.

## Methods

### Synthesis

The target material was synthesized by reacting 1.54 g Na_2_SO_4_ (Wako, 99%) and 2.73 g FeSO_4_. The anhydrous FeSO_4_ precursor was prepared in-house by annealing commercial FeSO_4_.7H_2_O (Wako, 99%) under vacuum at 200 °C for 12 h (ref. 35). Na_2_Fe_2_(SO_4_)_3_ cathode compound was obtained via classical solid-state synthesis by ball milling the precursors for 4 h followed by annealing the mixture at 350 °C for 24 h under steady Ar flow. As SO_4_-based compounds are prone to dissolvation (in water) and thermal decomposition, we used these sustainable non-aqueous, low-temperature methods. Chemical oxidation was performed to obtain desodiated Na_2−*x*_Fe_2_(SO_4_)_3_ samples using NO_2_BF_4_ (Alfa Aesar, 96%) oxidant dissolved in acetonitrile solvent (Wako, H_2_O level <5 p.p.m.). The solution was stirred overnight (with steady Ar flow), and the final products were filtered and dried at 60 °C under vacuum.

### Material characterization

X-ray powder diffraction patterns were acquired in the 2*θ* range of 10–80° by a Bruker AXS D8 ADVANCE powder diffractometer equipped with a Co Kα radiation source operating at 35 kV and 40 mA. Synchrotron powder XRD data for Rietveld refinement was obtained under vacuum at the BL-4B_2_ beam line of Photon Factory (PF), High Energy Accelerator Research Organization (KEK), Tsukuba, Japan. The wavelength was calibrated to be 1.196179(10) Å. For all the XRD measurements, samples were mounted on an air-tightened custom-designed sample holder, which was covered with polyimide film inside an Ar-filled glove box to avoid any undesirable influence of air exposure. The determination of the peak positions and indexing were carried out with TOPAS-Academic Ver. 4.1 programme. The structure of Na_2_Fe_2_(SO_4_)_3_ was solved by the parallel tempering algorithm^37^ available in the global optimization programme FOX^38^, where tetrahedral constraints are applied to SO_4_ units. The positions and occupancies were refined by subsequent Rietveld refinement using TOPAS-Academic Ver.5 programme, and the final structure was illustrated with VESTA software^39^. The BVS for Na is calculated for whole space in the unit cell of Na_2_Fe_2_(SO_4_)_3_ within a grid resolution of 0.1 Å. The modified ‘soft-BV’ parameters are used by utilizing an expanded evaluation range of the bonding interaction; *r*_0_=1.5602 and *B*=0.483 for Na–O bond^28^. The penalty term of asymmetric coordinate was neglected. The Mössbauer spectra were taken with a Topologic System Inc. spectrometer with a ^57^Co γ–ray source, calibrated with α–Fe as standard. The model fitting was performed with MossWinn 3.0 software. Particle morphology of powder samples was analysed by a Hitachi S-4800 field-emission scanning electron microscope operating at 2 kV.

### Electrochemical characterization

For electrochemical measurements, the working electrode was formulated by mixing 80 wt% Na_2_Fe_2_(SO_4_)_3_ active material, 15 wt% carbon black (Ketjen Black ECP, Lion Corp.) and 5 wt% polytetrafluoro-ethylene binder. This working electrode tape was pressed on an Ti mesh working as the current collector, with an average cathode loading of ca. 3 mg cm^−2^. Beaker-type three-electrode cells were assembled inside an Ar-filled glove box by taking the cathode film as the working electrode and Na metal foils acting as counter and reference electrodes. These beaker cells were filled with 1 M NaClO_4_ dissolved in propylene carbonate acting as electrolyte with no additives. Galvanostatic charge–discharge cycling was conducted in the voltage ranges, 2.0–4.5 V, at different rates from C/20 to 20C (at 25 °C). Rate capability tests were carried out using 2032-type coin cells with Na metal anode. Composite positive electrodes of 85 wt% active materials, 10 wt% ECP and 5 wt% polyvinylidene fluoride were mixed in N-methylpyrrolidone. The slurry was uniformly casted on an Al foil with an average loading of *ca*. 1 mg cm^−2^, and dried at 120 °C under vacuum. The electrolyte solution was 1.0 mol dm^−3^ NaPF_6_ dissolved in a mixture of ethylene carbonate:diethyl carbonate (5:5 by vol., Kishida Chemical) with 2 vol% of fluorinated ethylene carbonate (Kishida Chemical) as an electrolyte additive^40^. A glass fibre filter (GB-1000R, ADVANTEC) was used as a separator. The coin cells were discharged to 1.5 V at different rate from C/20 to 20C. Before each discharge, the cells were charged at C/20 to 4.2 V.

### *In situ* XRD

*In situ* XRD measurements were conducted on BL-3A at KEK-PF using synchrotron radiation (*λ*=0.12 nm) at room temperature. Diffraction data (exposure time, 30 s) were collected with reflection geometry by a two-dimensional detector (PILATUS-100K, DECTRIS). As an electrode, 80 wt% Na_2_Fe_2_(SO_4_)_3_, 10 wt% ECP and 10 wt% polytetrafluoro-ethylene binder were mixed, pressed onto a 10 μm Al foil and dried at 120 °C under vacuum. An *in situ* XRD cell (RIGAKU) filled with 1 M NaClO_4_ dissolved propylene carbonate electrolyte was assembled in the following order: Be window, Na_2_Fe_2_(SO_4_)_3_ pellet on Al foil, a glass fibre filter and Na metal. The cell was cycled in the voltage range of 2–4.2 V at C/5 current rate (at 25 °C).

### Computation methods

All the density functional theory (DFT) calculations were performed with the Vienna *ab initio* simulation package VASP^41^. The projector augmented wave method^42^ as implemented in the VASP code was used. The generalized gradient approximation (GGA) (PBE^43^) exchange-correlation functional was assumed, this eliminated the contribution of polaron transport in the migration calculations when the GGA+*U* method was used and allowed comparison with literature values. Spin-polarized calculations assuming ferromagnetic orderings of the Fe ions were conducted. A cutoff of 520 eV for the planewave expansion was used. The integration in the reciprocal space was conducted at the Γ point. Both Na poor and rich regions were investigated and was modelled with a 1 × 1 × 2 supercell that has a formula of Na_1_Fe_16_S_24_O_96_ per unit cell containing 137 atoms. The activation energy for Na^+^ migration was calculated with the climbing nudged elastic band^44^ method. The unit cell dimensions were fixed at the optimized unit cell size of this concentration during the nudged elastic band runs, otherwise all the ions were allowed to relax. Convergence of the forces were set to 0.015 eV Å^−1^ and the equilibrium pressure was smaller than |0.5| kbar.

## Author contributions

G.O. carried out most of the experimental work on synthesis, structural, physical and electrochemical characterization following some initial work by P.B.; S.N. conducted all crystal structure and diffraction pattern analyses; and S.-C.C. performed the theoretical calculation. The ideas and experiments were conceived, analysed and planned by all co-authors led by A.Y. The manuscript was written by all authors under the supervision of A.Y.

## Additional information

**How to cite this article**: Barpanda, P. *et al*. A 3.8-V earth-abundant sodium battery electrode. *Nat. Commun*. 5:4358 doi: 10.1038/ncomms5358 (2014).

## Supplementary Material

Supplementary Information Supplementary Figures 1-3 and Supplementary Tables 1-5
