# Supplementary material for: A 3.8-V earth-abundant sodium battery electrode
Source: Nat Commun. 2014 Jul 17;5:4358. doi: 10.1038/ncomms5358 (PMC4109020; doi:10.1038/ncomms5358)
Supplement: Supplementary Information — Supplementary Figures 1-3 and Supplementary Tables 1-5 [file ncomms5358-s1.pdf]

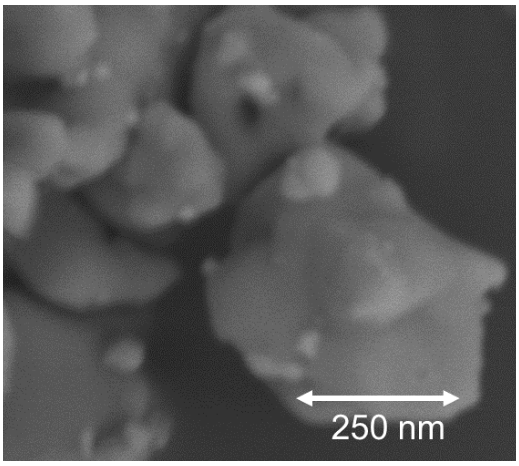

Supplementary Figure 1: A representative SEM micrograph of synthesized $Na_2Fe_2(SO_4)_3$ particles.

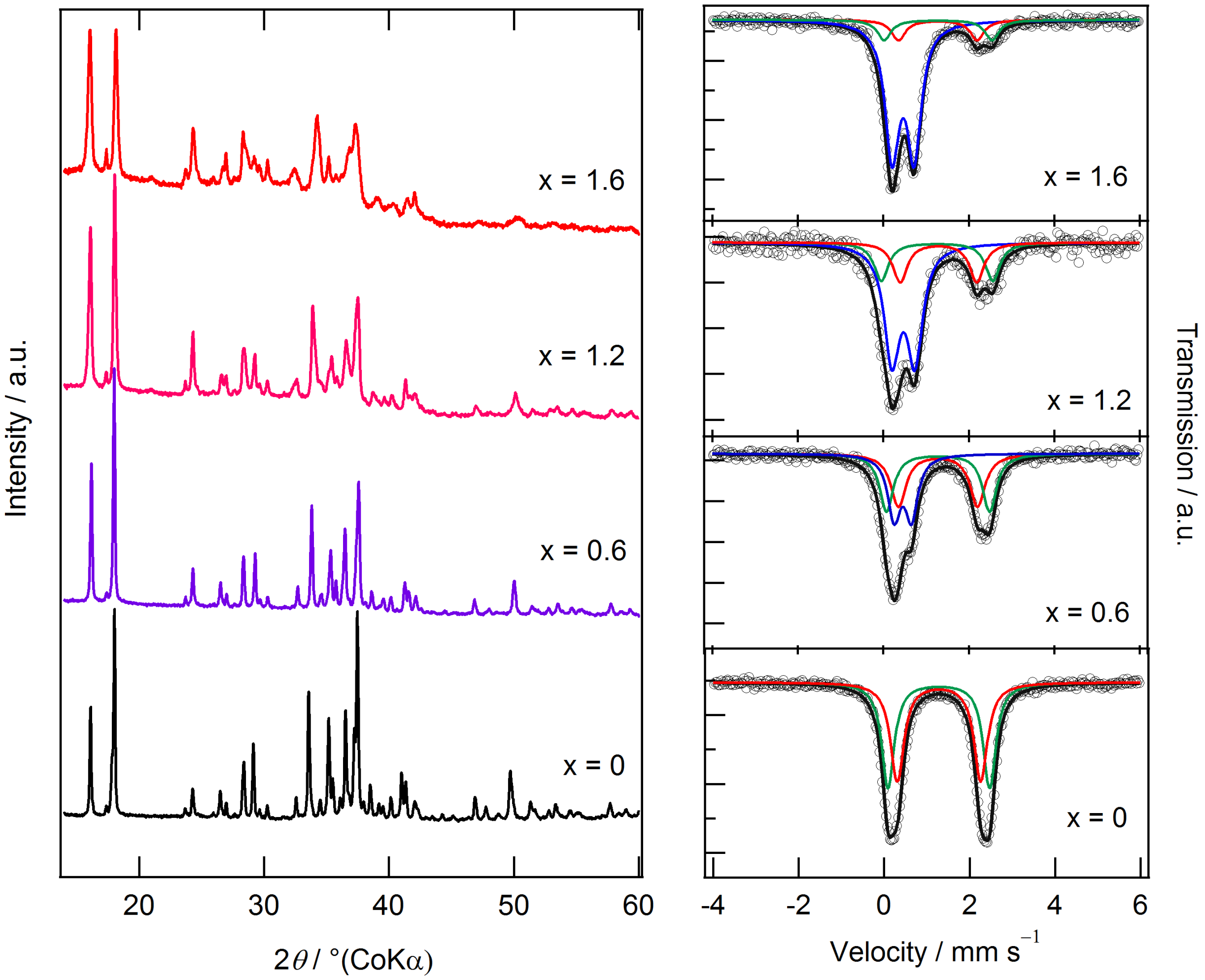

Supplementary Figure 2: X-ray diffraction patterns and Mössbauer spectra of the $Na_{2-x}Fe_2(SO_4)_3$ solid solution (x = 0, 0.6, 1.2, and 1.6) prepared by chemical oxidation.

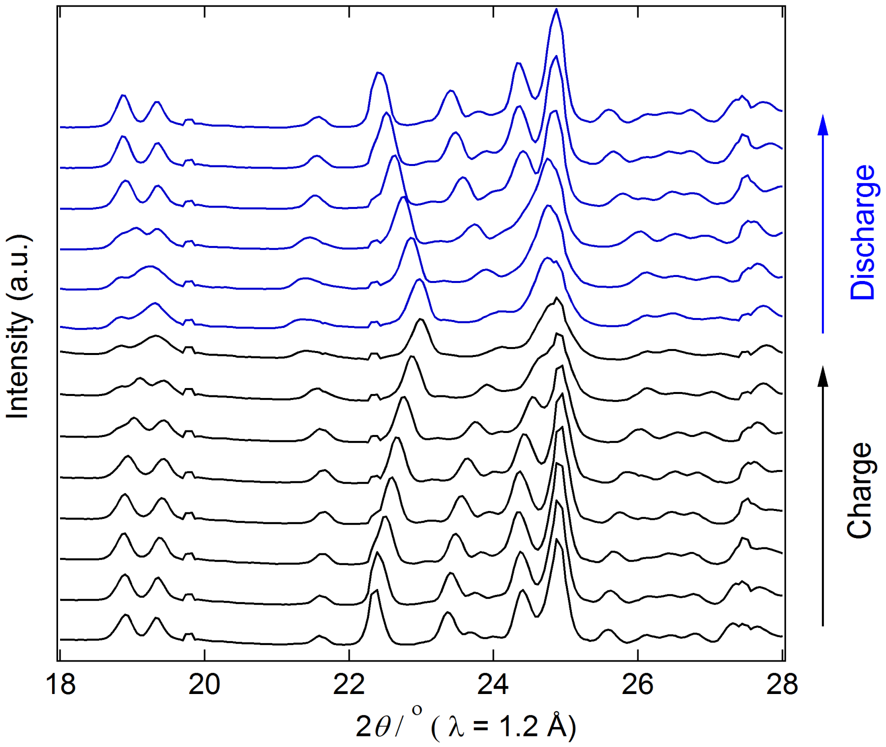

Supplementary Figure 3: In-situ X-ray diffraction patterns of the $Na_{2-x}Fe_2(SO_4)_3$ during electrochemical charge and discharge at a rate of C/5 (3rd cycle). The reaction is dominated by the single-phase reaction. Peak splitting (two to three) at 18-19 degree is a result of anisotropic lattice deformation. Small peak at 22.2 degree is a detector noise. Therefore, they do not represent the appearance of second phase.

Supplementary Table 1: Crystallographic data of $Na_{2+\delta}Fe_{2-\delta/2}(SO_4)_3$ with $P2_1/c$ space group

Chemical formula	$Na_{2.263}Fe_{1.868}(SO_4)_3$
M_r	444.36(7)
Crystal System, spece group	Monoclinic, $P2_1/c$ (No.14)
Temperature	~ 298 K (ambient)
a, b, c (Å)	11.46967 (9), 12.77009 (10), 6.51177 (5)
α, β, γ (°)	90, 95.2723 (5), 90
V (Å^3)	949.74 (11)
Z	4
True density (g/cm^3)	3.1077(5)
R_{wp}	0.0487
R_p	0.0394
GoF	1.74
R_{Bragg}	0.0158
Radiation type	Synchrotron, λ = 1.196179(10) Å

Supplementary Table 2: Fractional atomic coordinates, occupancies and isotropic displacement parameters for $P2_1/c$ model

Site	Wyckoff	x / a	y / b	z / c	occ.	B / Å^2
Na1	4e	0.2453 (10)	0.5156 (2)	0.5065 (17)	1.0	2.96 (7)
Na2	4e	0.7463 (15)	0.2424 (11)	0.266 (4)	0.741 (5)	7.03(9)
Na3	4e	0.2393 (12)	0.2365 (5)	0.465 (2)	0.563 (15)	4.73(9)
Fe1	4e	0.4832 (2)	0.0897 (2)	0.3356 (5)	0.925 (5)	1.34 (7)
Fe2	4e	0.0215 (3)	0.4052 (2)	0.1671 (5)	0.923 (5)	2.09 (8)
S1	4e	0.0129 (5)	0.1433 (3)	0.1434 (9)	1.0	1.91 (3)
O11	4e	0.8955 (8)	0.1728 (6)	0.0655 (14)	1.0	2.09 (4)
O12	4e	0.0872 (6)	0.0927 (8)	0.9989 (14)	1.0	= O11
O13	4e	0.0167 (10)	0.0740 (7)	0.3352 (19)	1.0	= O11
O14	4e	0.0616 (7)	0.2519 (8)	0.1892 (16)	1.0	= O11
S2	4e	0.4884 (5)	0.3498 (4)	0.3583 (9)	1.0	= S1
O21	4e	0.4849 (10)	0.4149 (8)	0.1684 (19)	1.0	= O11
O22	4e	0.4226 (7)	0.2569 (8)	0.2992 (16)	1.0	= O11
O23	4e	0.4367 (6)	0.4089 (8)	0.5165 (15)	1.0	= O11
O24	4e	0.6144 (8)	0.3493 (6)	0.4441 (14)	1.0	= O11
S3	4e	0.2477 (6)	0.52719 (15)	0.9942 (12)	1.0	= S1
O31	4e	0.1612 (8)	0.6014 (7)	0.8906 (15)	1.0	= O11
O32	4e	0.2018 (8)	0.4604 (8)	0.1581 (17)	1.0	= O11
O33	4e	0.3119 (9)	0.4570 (8)	0.8609 (17)	1.0	= O11
O34	4e	0.3287 (8)	0.5900 (7)	0.1259 (15)	1.0	= O11

Supplementary Table 3: Crystallographic data of $Na_{2+\delta}Fe_{2-\delta/2}(SO_4)_3$ with *C2/c* space group

Chemical formula	$Na_{2.256}Fe_{1.872}(SO_4)_3$
M_r	444.57(2)
Crystal System, spece group	Monoclinic, *C*2/*c* (No.15)
Temperature	~ 298 K (ambient)
a, b, c (Å)	12.65847 (7), 12.77062 (7), 6.51210 (3)
α, β, γ (°)	90, 115.5391 (4), 90
V (Å^3)	949.86 (11)
Z	4
True density (g/cm^3)	3.10880(17)
R_{wp}	0.0488
R_p	0.0400
GoF	1.74
R_{Bragg}	0.0189
Radiation type	Synchrotron, λ = 1.196179(10) Å

Supplementary Table 4: Fractional atomic coordinates, occupancies and isotropic displacement parameters for *C*2/*c* model

Site	Wyckoff	x/a	y/b	z/c	occ.	B / Å^2
Na1	4*e*	1/2	0.7333 (2)	3/4	1.0	2.75(6)
Na2	4b	0	0	0	0.741 (5)	6.16(16)
Na3	4*e*	1/2	0.9874 (4)	1/4	0.563 (15)	4.87(19)
Fe1	8*f*	0.73096 (6)	0.15796 (6)	0.14721 (13)	0.9359 (13)	1.847 (19)
S1	4*e*	0	0.77639 (13)	3/4	1.0	1.81 (4)
O11	8*f*	0.0844 (2)	0.8456 (2)	0.7181 (4)	1.0	1.87(6)
O12	8*f*	0.4457 (2)	0.20944 (18)	0.5469 (4)	1.0	1.58(6)
S2	8*f*	0.76214 (11)	0.60305 (9)	0.8696 (2)	1.0	1.81(3)
O21	8*f*	0.76555 (18)	0.66917 (19)	0.6841 (4)	1.0	1.09(6)
O22	8*f*	0.3198 (2)	0.9958 (2)	0.3765 (4)	1.0	2.44(6)
O23	8*f*	0.3601 (2)	0.5869 (2)	0.6708 (5)	1.0	2.64(8)
O24	8*f*	0.3252 (2)	0.1578 (2)	0.0837 (5)	1.0	2.19(6)

Supplementary Table 5: Refined Mössbauer spectrum parameters of $Na_{2-x}Fe_2(SO_4)_3$ solid solution phases.

x in $Na_{2-x}Fe_2(SO_4)_3$		Isomer shift / $mm\ s^{-1}$	Quadrupole splitting / $mm\ s^{-1}$	Fraction / %	Line width / $mm\ s^{-1}$
0	Fe^{2+}(1)	1.2798 (8)	2.378 (3)	50	0.356 (3)
	Fe^{2+}(2)	1.2798 (8)	1.954 (3)	50	0.382 (4)
	Fe^{3+}(3)	-	-	-	-
0.6	Fe^{2+}(1)	1.281 (4)	2.411 (7)	33.6 (4)	0.427 (1)
	Fe^{2+}(2)	1.281 (4)	1.852 (9)	33.6 (4)	0.389 (8)
	Fe^{3+}(3)	0.457 (3)	0.408 (8)	32.7 (5)	0.356 (8)
1.2	Fe^{2+}(1)	1.283 (1)	2.63 (3)	20.1 (3)	0.423 (2)
	Fe^{2+}(2)	1.283 (1)	1.81 (2)	20.1 (3)	0.433 (2)
	Fe^{3+}(3)	0.468 (5)	0.542 (9)	59.9 (9)	0.446 (1)
1.6	Fe^{2+}(1)	1.277 (8)	2.52 (2)	11.5 (4)	0.38 (1)
	Fe^{2+}(2)	1.277 (8)	1.83 (2)	11.5 (4)	0.38 (2)
	Fe^{3+}(3)	0.463 (1)	0.517 (3)	77.0 (6)	0.398 (4)

Normalized χ^2 for each fitting were 1.34, 1.22, 1.04 and 1.02 for x = 0, 0.6, 1.2, and 1.6, respectively.
